# Nature and prevalence of long-term conditions in people with intellectual disability: retrospective longitudinal population-based study

**DOI:** 10.1136/bmjopen-2024-090857

**Published:** 2025-01-22

**Authors:** Gemma Lewin, Rania Kousovista, Emeka Abakasanga, Rishika Shivamurthy, Georgina Cosma, Gyuchan Jun, Navjot Kaur, Ashley Akbari, Satheesh Gangadharan

**Affiliations:** 1 Leicestershire Partnership NHS Trust, Leicester, UK; 2 Department of Computer Science, Loughborough University, Loughborough, UK; 3 Leicester Centre for Mental Health Research, Leicestershire Partnership NHS Trust, Leicester, UK; 4 Loughborough University Loughborough School of Design and Creative Arts, Loughborough, UK; 5 Leicester Real World Evidence Unit, Diabetes Research Centre, University of Leicester, Leicester, UK; 6 Swansea University Medical School, Swansea, UK

**Keywords:** Multimorbidity, PSYCHIATRY, Chronic Disease, EPIDEMIOLOGY, EPIDEMIOLOGIC STUDIES, MENTAL HEALTH

## Abstract

**Abstract:**

**Objective:**

Explore the nature and prevalence of long-term conditions in individuals with intellectual disability.

**Design:**

Retrospective longitudinal population-based study.

**Setting:**

Primary and secondary care data across the population of Wales with the Secure Anonymised Information Linkage (SAIL) Databank.

**Participants:**

14 323 individuals were identified during the study date period 1 January 2000 to 31 December 2021 using the following inclusion criteria: 18 or older, alive at the cohort start date, a resident of Wales, with a primary care registration at a SAIL providing general practice with available records and a recorded diagnosis of intellectual disability. Once individuals were identified, health records were observed from birth.

**Results:**

13 069 individuals had a recorded diagnosis of intellectual disability and at least one long-term condition, reflecting 91.25% of the population. Demographic data from the SAIL dataset reveal that the study population is predominantly White, with low levels of representation of non-White ethnic groups. In the cohort, a larger proportion of patients live in the most deprived areas of Wales (22.30%), with fewer individuals in less deprived categories. Mental illness was identified as the most prevalent of the identified long-term conditions, whereby 30.91% of the population had a recorded diagnosis of a mental illness which was chronic. For many common conditions, including epilepsy, thyroid disorders, upper gastrointestinal disorders, chronic kidney disease and diabetes, there was an overall trend of higher prevalence rates in the intellectual disability cohort when compared with the general population. The prevalence of hypertension was lower in individuals with intellectual disability. Chronic constipation, chronic diarrhoea and insomnia were examples of long-term conditions added as relevant to individuals with intellectual disability. Notable differences in the distribution of long-term conditions were observed when comparing across sex and age groups. The number of long-term conditions increases with age. Conditions which may usually be expected to emerge later in life are present in younger age groups, such as diabetes, hypertension and chronic arthritis. When hospital episodes were analysed, epilepsy, diabetes, chronic airway disease and mental illness were commonly treated conditions during hospital admission across both sexes. Conditions which were less prevalent in the intellectual disability cohort, but which were treated during ≥6% of total hospital admissions include cancer, cardiac arrhythmias and cerebral palsy.

**Conclusions:**

This study establishes a range of 40 relevant long-term conditions for people with intellectual disability through an iterative process, which included a review of the available literature and a series of discussions with a Professional Advisory Panel and Patient and Public Involvement groups of this research project. The findings of the study reinforce the high prevalence and early emergence of long-term conditions in the intellectual disability cohort. It also demonstrates the difference in the range of conditions when compared with the general population. There were differences in long-term conditions when separated by sex and age. Long-term conditions which commonly require treatment in hospitals were also revealed. Further work is required to translate the findings of this study into actionable insights. Clusters of multiple long-term conditions, trajectories, outcomes and risk factors should be explored to optimise the understanding and longitudinal care of individuals with intellectual disabilities and long-term conditions.

**STRENGTHS AND LIMITATIONS OF THIS STUDY**

- This study is a population-scale, anonymised, individual-level longitudinal cohort of 14 323 participants with intellectual disability, which strengthens the generalisability of the reported findings.
- The Secure Anonymised Information Linkage dataset encompasses primary and secondary care data, which facilitates representation across the spectrum of intellectual disability, across a wide set of healthcare settings.
- This study establishes a range of relevant long-term conditions for people with intellectual disability using an iterative process which included a review of the literature and a series of discussions with Professional Advisory Panel and Patient and Public Involvement groups who offer invaluable expertise and have identified long-term conditions which are impactful on the lives of people with intellectual disability.
- The iterative process does not use a robust methodology such as a Delphi review, which is a noted limitation of the study.
- Using routinely collected data sources which capture records using coding classifications such as Read codes and International Classification of Diseases version 10 codes reduces participation bias but can present limitations relating to data quality and variability in usage.

## Introduction

 Intellectual disability constitutes a common neurodevelopmental disorder marked by considerable heterogeneity.^1^ Operationally defined by the World Health Organisation as a state of arrested or incomplete development of the mind, intellectual disability manifests with discernible impairments across domains crucial to overall intelligence, including cognitive, language, motor and social capacities.^2^ Global estimates of intellectual disability prevalence are around 1%.^3^

Individuals with intellectual disability experience a higher vulnerability to long-term conditions and multiple long-term conditions (MLTC), a term which is defined as the presence of two or more long-term health conditions concurrently.^4^ There is an elevated prevalence of MLTC in individuals with intellectual disability, with one large population-based study revealing a mean of 11 conditions, and an overwhelming prevalence of MLTC at 98.7%.^5^ Additionally, MLTC appears to have an earlier age of onset in people with intellectual disability. For example, prevalence rates of MLTC are similar for people aged 20 to 25 in individuals with intellectual disability, when compared with people aged 50 to 54 in the general population.^6^ Unsurprisingly, this disease burden contributes to premature mortality, polypharmacy and poor quality of life.^7 8^

In addition to the high prevalence and earlier onset, individuals with intellectual disability exhibit a distinct pattern of long-term conditions, different from those of the general population.^9^ Conditions such as epilepsy, gastro-oesophageal reflux disorder, chronic constipation and sensory impairments are markedly more prevalent among this population. One-third to half of people with intellectual disability experience chronic constipation which contributes to morbidity and premature mortality.^10^ Individuals with intellectual disability are more prone to diabetes, almost three times more susceptible to arthritis, and over twice as likely to develop cardiovascular disease and asthma.^11^

In addition to summarising the evidence supporting differences in the pattern of long-term conditions, increased prevalence, a higher number of co-occurring conditions and earlier onset of MLTC when compared with the general population, a recent scoping review of literature on long-term conditions in people with intellectual disability by Mann *et al*^12^ also highlighted the limitations of published work. A substantial proportion of the literature was focused on subgroups within the intellectual disability population, such as people with Down syndrome or specific age groups. The population studied varied from patients attending specialist clinics to patients identified using disease registers. Only a small number of studies have used the full population. Most studies are limited by their sample size, with only two studies that had a sample size of over 5000. These limitations could lead to a conclusion on long-term conditions in the intellectual disability population which is not representative of the heterogeneous population.

One way to overcome these limitations is to use a population-scale database which covers both primary and secondary care records. As the UK is an early adopter of electronic patient records, these databases provide a wealth of data on the health needs of the population. This study is novel in that it aims to provide comprehensive information regarding the nature and prevalence of long-term conditions in individuals with intellectual disability, as identified from a population-scale databank which covers the scope of Wales.

This paper is part of a research project entitled Data-driven machinE-learning aided stratification and management of multiple long-term COnditions in adults with intellectual disabilitiEs (DECODE). DECODE is a National Institute for Health and Care Research-funded research project which aims to apply machine learning approaches to identify clusters and trajectories of long-term conditions in people with intellectual disabilities, and to use them to develop actionable insights and practical usage scenarios for effective care coordination to improve the health and well-being of people with intellectual disabilities. Machine learning algorithms are used to identify the clusters of MLTC in people with intellectual disability, including trajectories, interactions with risk factors, and outcomes. The information covered in this paper is not analysed using machine learning algorithms.

## Methods

### Study design

The study design is a retrospective longitudinal population-based study. We used the Secure Anonymised Information Linkage (SAIL) Databank to identify a population representative of individuals with intellectual disability. To identify the presence of intellectual disability, Read codes from primary care records were used. These codes included codes referring to the presence of intellectual disability or genetic syndromes suggesting the presence of intellectual disability (eg, Lesch-Nyhan syndrome). A list of Read codes was prepared using previously used repositories,^13^ which was then further developed and finalised by a Professional Advisory Panel (PAP) which included two consultant psychiatrists specialised in intellectual disability and two general practitioners. 14 323 individuals were identified during the study date period 1 January 2000 to 31 December 2021 using the following inclusion criteria: 18 or older, alive at the cohort start date, a resident of Wales, with a primary care registration at a SAIL providing general practice with available records and a recorded diagnosis of intellectual disability. Once individuals were identified, health records were observed from birth.

### Data acquisition and data preparation

We obtained data for this study from the SAIL Databank to identify the nature and prevalence of long-term conditions in an intellectual disability cohort from the population of Wales, and the associated demographic and socioeconomic characteristics of the study cohort. The SAIL Databank is the national Trusted Research Environment for Wales and holds anonymised, longitudinal individual-level, population-scale data linkable data sources, comprising billions of anonymised records and reflecting the patient journey of millions of individuals in Wales.^14^ SAIL uses an Anonymised Linking Field with encryption to ensure that data can be analysed longitudinally through primary care general practice, secondary care (including emergency department, hospital inpatient and hospital outpatient) and mortality data sources.^15^ Primary care data are captured in Read codes, are available from the Welsh Longitudinal General Practice data and contain diagnosis, medication and process-of-care events. Hospital data are captured in International Classification of Diseases version 10 (ICD-10) codes, are available from the Patient Episode Database for Wales data and contain diagnosis, admission and discharge admissions. Death data are available from the Annual District Death Extract and from the Office for National Statistics mortality register and use the ICD-10 codes. The Welsh Demographic Service Dataset was used to capture individuals’ residency history and associated demographic and socioeconomic characteristics.

Research using routinely collected data sources needs an approach for data interpretation supported by clinicians who are normally involved in recording these data. Therefore, this project used a PAP comprising a multidisciplinary team with relevant expertise, including general practitioners, a consultant psychiatrist specialised in intellectual disability, nurses, pharmacists and data analysts to support the extraction and interpretation of appropriate clinical codes in the data.

A recent study established the list of long-term conditions as relevant to the general population for studies on MLTC.^16^ Given the distinct differences in the nature of long-term conditions applicable to individuals with intellectual disability, this study developed a list of 40 conditions using a combination of a review of the literature and a review by the PAP. A potential list of long-term conditions was initially formulated using a scoping review of the literature,^12^ which was reviewed and revised by the PAP. Read codes and ICD-10 codes were mapped to the list of conditions. The PAP was able to interpret the suitability of codes so that each condition was accurately reflected.

Suitable chronic conditions were combined following clinical discussion and consensus; for example, clinical codes of recurrent depression, bipolar disorder, chronic anxiety and schizophrenia were included for the mental illness category, excluding conditions like a single episode of depression or acute and transient mental health conditions. The mental illness category did not include behavioural disturbances. Atrial fibrillation, atrial flutter, supraventricular tachycardia and heart block were included in the category of cardiac arrhythmias; hyperthyroidism, hypoparathyroidism and Hashimoto’s disease under thyroid disorders; fibromyalgia, pelvic pain and chronic back pain under chronic pain conditions; gastro-oesophageal reflux disease, gastric and peptic ulcer diseases, *Helicobacter pylori* and dyspepsia under upper gastrointestinal disorders; chronic eosinophilic bronchitis, chronic obstructive pulmonary disease (COPD) and asthma under chronic airway diseases; and osteoarthritis, arthritis and autoimmune arthritis under chronic arthritis. Following discussion with the PAP, conditions where less than five counts were identified within the dataset were removed. Through iterative review and revision as described above, we identified a list of 40 long-term conditions which are relevant for individuals with intellectual disability.

We identified long-term conditions by considering a definition of chronicity for each condition. Patients with a chronic code description (Read code or ICD-10 code) for the specific conditions were considered to have chronic conditions and were included in the analysis even if the code was used only once in the data. In cases lacking a chronic code description, duration criteria based on definitions in literature and analyses are applied: at least two code records with more than n-days apart (based on duration cited in relevant literature for each condition) within the timeframe of a year. For constipation and back pain, a period of greater than 3 months between the records was selected, aligning with definitions in the literature.^17 18^ Diarrhoea was considered as chronic if at least two records are more than a month apart in duration.^19 20^ Pneumonia is defined as chronic pneumonia due to any microorganisms which persist for 6 weeks or longer.^21^ This part of the work considered evidence from the literature and the views of clinicians in the PAP when determining the duration of chronicity for each condition.

### Study sample

**Figure 1:**
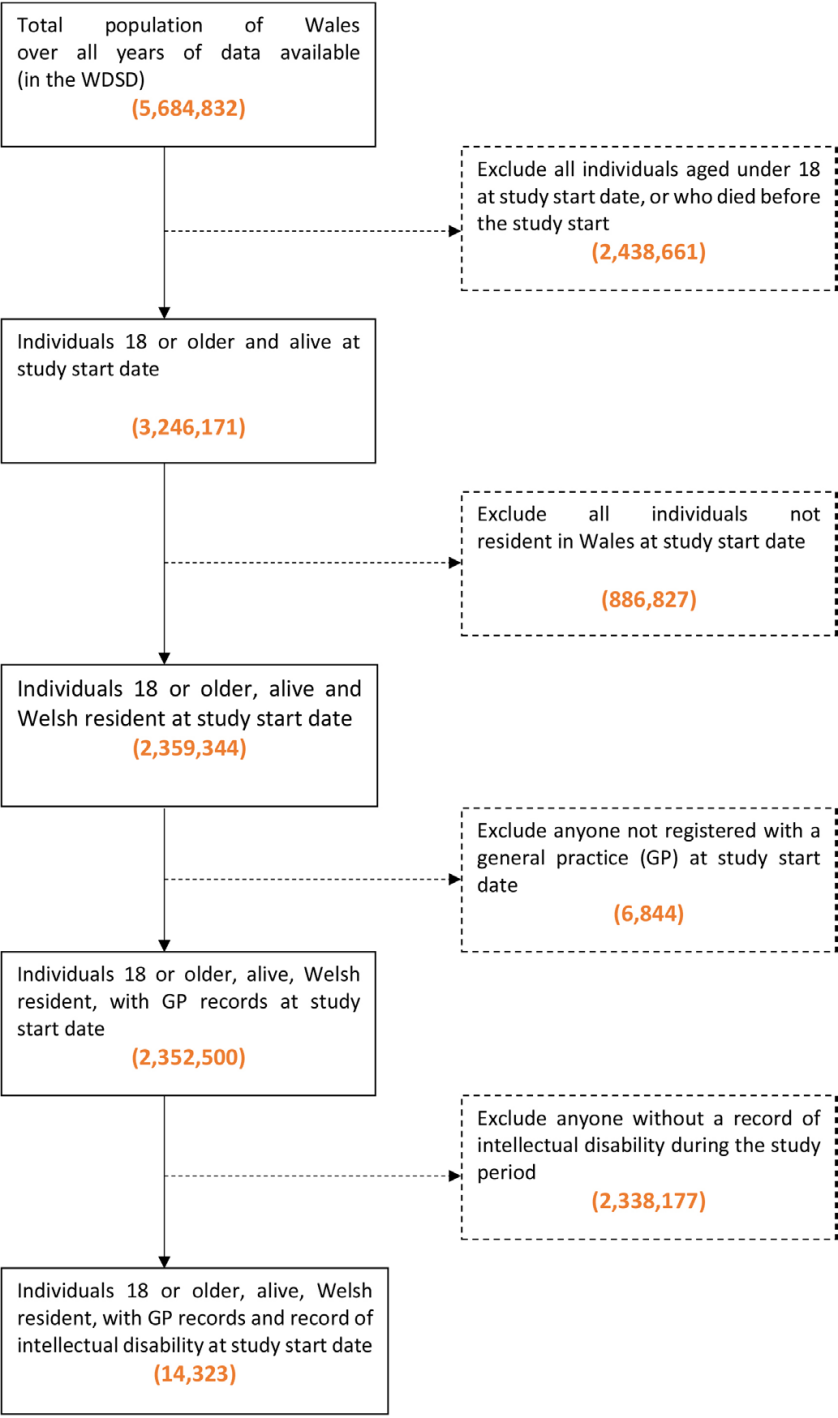
Flow diagram with inclusion and exclusion criteria for the study cohort.

14 323 individuals were identified during the study date period 1 January 2000 to 31 December 2021 using the following inclusion criteria: 18 or older, alive at the cohort start date, a resident of Wales, with a primary care registration at a SAIL providing general practice with available records and a recorded diagnosis of intellectual disability (figure 1). Once individuals were identified, health records were observed from birth. Subgroups of sex and age were used to observe whether there were differences to note between groups.

### Data analysis

Descriptive calculation methods, such as percentages and frequencies, were used for the data analysis in the study and presented in this paper. Where prevalence is calculated, it is reflective of lifetime prevalence (the proportion of individuals who have experienced the characteristic of interest since birth up to the time of the assessment).

### Patient and Public Involvement

The DECODE project is supported by a Patient and Public Involvement (PPI) panel, which consists of three groups of experts by experience: people with intellectual disability, carers of people with intellectual disability, and professional carers of people with intellectual disability. People with intellectual disability received training on being a PPI member in research projects.^22^ In addition, a learning disability nurse and speech and language therapist supported the PPI groups, with accessible information and any additional support for the individuals to aid understanding and participation. A considerable proportion of the PPI group had a diagnosis of a long-term condition or cared for someone with a long-term condition. The PPI group worked with researchers to ensure that a list of long-term conditions are relevant to the study population.

## Results

### Demographics

The demographic characteristics of individuals included in the study were recorded (table 1). The population reflects the characteristics of adults with intellectual disability in the data. Individuals are predominantly White (68.22%), with 29.68% of individuals having an unknown ethnic group. Within the cohort, ethnic minority groups were not well represented and were reported as Asian (1.33%), Black (0.26%) and mixed/other (0.5%).

**Table 1:** Characteristics of adults with intellectual disability

Characteristics	N	N %
Patients	14 323	100
Sex		
Female	6652	46.44
Male	7671	53.56
Age (years)		
<20	751	5.24
20–29	2433	16.99
30–39	3459	24.15
40–49	3393	23.69
50–59	2363	16.50
60–69	1223	8.54
70–79	551	3.84
80+	150	1.05
Ethnic groups		
White	9772	68.22
Asian	192	1.33
Black	37	0.26
Mixed/other	71	0.50
Unknown	4251	29.68
WIMD		
1 (most deprived)	3195	22.30
2	2582	18.03
3	2087	14.57
4	1949	13.61
5 (least deprived)	1346	9.40
LTC ≥1	13 069	91.25

LTC, long-term conditionWIMDWelsh Index of Multiple Deprivation

The socioeconomic status of patients was characterised using the 2019 Welsh Index of Multiple Deprivation (WIMD), which is a measure of deprivation in Wales.^23^ For the purpose of this study, the WIMD scores were grouped into five categories, ranging from 1 (representing the most deprived areas) to 5 (representing the least deprived areas). A larger proportion of patients fall into the most deprived category (22.30%), with fewer individuals in less deprived categories. 13 069 individuals had a recorded diagnosis of intellectual disability and at least one long-term condition, reflecting 91.25% of the population.

### Nature and prevalence of long-term conditions

Of the 13 069 individuals that had a recorded diagnosis of intellectual disability and at least one long-term condition, the number of individuals and the percentage of total individuals diagnosed with each of the 40 long-term conditions identified were calculated (table 2).

**Table 2:** Total distinct patient counts of each condition across general practice and hospital data

Condition	N	N %
Mental illness	4427	30.91
*Upper gastrointestinal disorders*	3903	27.25
Epilepsy	3886	27.13
Chronic airway disease	3296	23.01
Hypertension	3092	21.59
Thyroid disorders	2882	20.12
Chronic arthritis	2735	19.10
Chronic kidney disease	2655	18.54
Diabetes	2600	18.15
Anaemia	2238	15.62
Hearing loss	2200	15.36
*Insomnia*	2011	14.04
Cardiac arrhythmias	1665	11.63
*Dysphagia*	1636	11.42
Inflammatory bowel disease	1576	11.00
Chronic pain conditions	1568	10.95
Cancer	1446	10.10
Dementia	1326	9.26
Neuropathic pain	1309	9.14
*Menopausal and perimenopausal*	1189	8.30
Stroke	1150	8.03
Coronary heart disease	1148	8.01
*Chronic constipation*	1113	7.77
Osteoporosis	1103	7.70
*Cerebral palsy*	1063	7.42
Heart failure	1050	7.33
*Chronic diarrhoea*	871	6.08
Visual impairment	776	5.42
Peripheral vascular disease	699	4.88
*Psoriasis*	667	4.66
*Chronic pneumonia*	574	4.01
Parkinsons	202	1.41
*Barretts oesophagus*	161	1.12
Cirrhosis	135	0.94
Bronchiectasis	124	0.87
Interstitial lung disease	106	0.74
*Polycystic ovary syndrome*	56	0.39
*Tourette*	45	0.31
Addison’s disease	27	0.19
Multiple sclerosis	22	0.15

N is the absolute count of the cohort for each condition; N % is the proportion of the cohort with a specific condition compared tocompared with the total cohort population.

Conditions which differ from those defined by Ho *et al*^16^ are italicised.

Mental illness was identified as the most prevalent long-term condition, whereby 30.91% of the population had a recorded diagnosis of mental illness which was chronic.

The 10 most prevalent conditions include mental illness (30.91%), upper gastrointestinal disorders (27.25%), epilepsy (27.13%), chronic airway disease (23.01%), hypertension (21.59%), thyroid disorders (20.12%), chronic arthritis (19.10%), chronic kidney disease (18.54%), diabetes (18.15%) and anaemia (15.62%). Notably, there was a prevalence rate of over 15% for the 10 most prevalent conditions.

There were also some conditions included which are not present on the list of conditions defined by Ho *et al*.^16^ For example, chronic constipation, chronic diarrhoea and insomnia were added following review by the advisory panel and PPI group, as these were conditions which were felt to be relevant and important to the quality of the lives of individuals with intellectual disability. Conditions which differ from those defined by Ho *et al*^16^ are italicised within table 2.

To explore sex-specific trends, the 10 most prevalent long-term conditions for each sex were described (table 3). Sex was defined as male or female based on the first recorded instance of the patient’s intellectual disability within the study period. The number of patients and the percentage of patients within each sex group were recorded. Mental illness represents the most prevalent long-term condition for both females (32.89%) and males (29.19%). Some conditions are represented relatively equally between sexes, for example, upper gastrointestinal disorders (female 28.20%, male 26.42%), epilepsy (female 26.55%, male 27.64%), hypertension (female 22.40%, male 20.88%), chronic kidney disease (female 19.74%, male 17.49%) and diabetes (female 18.30%, male 18.03%).

**Table 3:** 10 most common long-term conditions across general practice and hospital data by sex

Females			Males		
Condition	N	N %	Condition	N	N %
Mental illness	2188	32.89	Mental illness	2239	29.19
Thyroid disorders	1921	28.88	Epilepsy	2120	27.64
Upper gastrointestinal disorders	1876	28.20	Upper gastrointestinal disorders	2027	26.42
Epilepsy	1766	26.55	Hypertension	1602	20.88
Chronic airway disease	1698	25.53	Chronic airway disease	1597	20.82
Hypertension	1490	22.40	Diabetes	1383	18.03
Chronic arthritis	1471	22.11	Chronic kidney disease	1342	17.49
Chronic kidney disease	1313	19.74	Chronic arthritis	1264	16.48
Anaemia	1285	19.32	Hearing loss	1140	14.86
Diabetes	1217	18.30	Insomnia	1078	14.05

N is the absolute count of the cohort for each condition by sex; N % is the proportion of the cohort with a specific condition compared tocompared with the total cohort population by sex.

However, there are some sex disparities, whereby thyroid disorders (28.88%) are common in females; however, they are not represented in the 10 most common conditions in males. Similarly, anaemia has been diagnosed in 19.32% of females; however, it is not represented in the 10 most common conditions in males. There are some disparities between prevalence rates in chronic airway diseases (female 25.53%, male 20.82%) and chronic arthritis (female 22.11%, male 16.48%).

The 10 most common long-term conditions by age group were described: subgroups comprised of individuals aged 40 years and under (≤40 years) and those above 40 years (>40 years) (table 4).

**Table 4:** 10 most common long-term conditions across general practice and hospital data by age

≤40 years			>40 years		
Condition	N	N %	Condition	N	N %
Mental illness	2669	31.50	Hypertension	1878	32.10
Epilepsy	2361	27.87	Mental illness	1758	30.05
Upper gastrointestinal disorders	2257	26.64	Chronic arthritis	1712	29.26
Chronic airway disease	1806	21.32	Chronic kidney disease	1693	28.94
Thyroid disorders	1433	16.91	Upper gastrointestinal disorders	1646	28.13
Diabetes	1220	14.40	Epilepsy	1525	26.06
Insomnia	1217	14.37	Chronic airway disease	1489	25.45
Hypertension	1214	14.33	Thyroid disorders	1449	24.77
Hearing loss	1059	12.50	Diabetes	1380	23.58
Chronic arthritis	1023	12.08	Anaemia	1251	21.38

N is the absolute count of the cohort for each condition by age; N % is the proportion of the cohort with a specific condition compared tocompared with the total cohort population by age.

There are conditions which are represented consistently through age groups, such as upper gastrointestinal disorders (≤40 years 26.64%, >40 years 28.13%) and chronic airway diseases (≤40 years 21.32%, >40 years 25.45%). In contrast, some conditions increase sharply, such as thyroid disorders (≤40 years 16.91%, >40 years 24.77%), diabetes (≤40 years 14.40%, >40 years 23.58%), hypertension (≤40 years 14.33%, >40 years 32.10%) and chronic arthritis (≤40 years 12.08%, >40 years 29.26%). Indeed, hypertension reflects the most common long-term condition in the >40 years group. There are also conditions which are represented only in the ≤40 years group, such as insomnia (14.37%) and hearing loss (12.50%). In the >40 years group, chronic kidney disease (28.94%) and anaemia (21.38%) are represented.

Hospital admission data between 2011 and 2021 were reported (table 5). There was a total of 17 587 female admissions and 18 541 male admissions. The absolute count represents an occurrence whereby a condition was treated during a hospital admission. Due to the multimorbid nature of the study population, multiple conditions may have required treatment during a single hospital admission. Percentage counts were reported which reflect the percentage of total admissions that required hospital treatment for the named condition. It should be noted that each condition is not necessarily the reason for the hospital admission.

**Table 5:** 10 most treated conditions during hospital admission by sex

Male			Female		
Condition	N	N %	Condition	N	N %
Epilepsy	5453	29.41	Epilepsy	4238	24.10
Diabetes	4519	24.37	Chronic airway diseases	4002	22.76
Chronic airway diseases	3619	19.52	Diabetes	3814	21.69
Mental illness	3252	17.54	Thyroid disorders	2995	17.03
Cancer	2243	12.10	Mental illness	2763	15.71
Chronic kidney disease	1933	10.43	Cancer	2575	14.64
Coronary heart disease	1783	9.62	Chronic kidney disease	2104	11.96
Thyroid disorders	1660	8.95	Chronic arthritis	1506	8.56
Cardiac arrhythmias	1465	7.90	Cardiac arrhythmias	1250	7.11
Cerebral palsy	1464	7.90	Cerebral palsy	1118	6.36

N is the absolute count whereby a condition was treated during a hospital admission in the cohort. N % is the percentage of total admissions that required hospital treatment for the named condition.

Epilepsy was the most treated condition during hospital admission across both sexes (male 29.41%, female 24.10%). Diabetes (male 24.37%, female 21.69%) and chronic airway diseases (male 19.52%, female 22.76%) were treated in a substantial number of hospital admissions. Mental illness (male 17.54%, female 15.71%) was treated during hospital admissions commonly; however, considering the high prevalence rates in the study group population, this is not surprising. Other conditions which are prevalent in the study group population and require treatment commonly in hospital are thyroid disorders (male 8.95%, female 17.03%) and chronic kidney disease (male 10.43%, female 11.96%).

There were numerous conditions which were often treated during hospital admission but were less prevalent in the study group population. Conditions included cancer (male 12.10%, female 14.64%), coronary heart disease (male 9.62%, female not reflected in table), cardiac arrhythmias (male 7.90%, female 7.11%) and cerebral palsy (male 7.90%, female 6.36%).

## Discussion

The findings of the study reinforce the astoundingly high prevalence of long-term conditions in the intellectual disability population (91.25%). In comparison, just under half of the UK population are reported to live with a long-term condition.^24^ The most prevalent long-term condition was mental illness (30.91%). The 10 most prevalent long-term conditions each had prevalence rates of over 15%, demonstrating the consistently high burden of common health conditions that were recorded within the cohort.

The difference in the range of conditions when compared with the general population is demonstrated. There were some stark differences, whereby epilepsy (27.13%) was markedly high when compared with the prevalence of epilepsy in the general population (0.76%).^25^ Similarly, thyroid disorders were far more prevalent (20.12%) when compared with the general population (3.82%).^26^ Other conditions which had a trend of higher prevalence rates in the intellectual disability population when compared with existing research in the general population included chronic mental illness,^27^ diabetes^28 29^ and chronic kidney disease.^30 31^ This contrasts with existing literature which observed similar prevalence of chronic kidney disease between both groups.^32^

There were conditions identified that had similar prevalence rates to the general population. Chronic airway diseases were reported in 23.01% of individuals in the study population. Rates are similar in the general population, whereby one in five people have a chronic airway disease.^33^ An increased prevalence of asthma and decreased prevalence of COPD has been reported in individuals with intellectual disability.^34^ The Learning Disability Mortality Review programme identified that individuals with intellectual disability are at increased risk of poor respiratory health, particularly relating to reduced quality of life and increased avoidable mortality rates.^35^ Chronic arthritis (19.10%) was reported at a similar rate to doctor-diagnosed chronic arthritis in the US (21.20%).^36^ Interestingly, rates of hypertension (21.59%) were lower than reported general population prevalence rates worldwide (31.1%).^37^ The literature varies, with some studies finding that those with intellectual disability have similar^38^,^41^ or decreased^42^ prevalence of hypertension.

Reported findings highlight age-specific and sex-specific trends and inform the need for a considered approach when determining management in this population. There is a shared burden of chronic diseases across sexes for individuals with intellectual disability; however, there were some sex disparities where females demonstrated a higher prevalence of thyroid disorders, anaemia, chronic airway disease and chronic arthritis.

It is also apparent that conditions which may usually be expected to emerge later in life are present in younger age groups for individuals with intellectual disability, such as diabetes, hypertension and chronic arthritis. A higher prevalence of long-term conditions was reported in the >40 years group. The emergence of different health conditions within the ≤40 and >40 years group reveals differing health priorities across age groups and highlights the shift in health concerns as individuals age. The data emphasise the need for age-specific healthcare strategies to address the dominant health challenges in each category.

Hospital admission data revealed the conditions treated most frequently during admission, and highlighted some conditions which were less prevalent in the cohort but contributed greatly to hospital treatment data, including cancer, coronary heart disease, cardiac arrhythmias and cerebral palsy. Secondary care data allow a wider picture to be built in order to understand the impact of long-term conditions, identifying potential areas for future research whereby additional surveillance and enhanced treatment packages can target findings across primary and secondary care.

Ho *et al*^16^ established a range of long-term conditions which were deemed as relevant for the general population and necessary to include in MLTC research. This consensus aims to achieve consistency and comparability and improve the quality of research related to MLTC. A literature review conducted by our team revealed that the existing range of identified conditions may not be appropriate to use in the heterogeneous intellectual disability population, where a number of conditions like epilepsy and constipation are unusually high.^12^ This approach establishes a range of relevant long-term conditions for people with intellectual disability, through an iterative process, which included a review of the literature and a series of discussions with PAP and PPI groups. Such groups have also been able to offer invaluable expertise to determine long-term conditions which are impactful on the lives of people with intellectual disability. The generated list of 40 conditions may have utility to use as a standard to optimise comparability and consistency within research relating to long-term conditions and people with intellectual disability.

The utilisation of information from a population-based sample including 14 323 participants is considerable. The cohort size is a marked strength of the study and supports the generalisability of the findings. It also facilitated a greater representation and breadth of individuals with intellectual disability. The utilisation of routinely recorded data across a broad spectrum of healthcare environments does not rely on individuals responding or actively participating in the study, which in turn can reduce participation bias. The use of anonymised data has enabled this to be completed with ethical consideration.

Although we have used an iterative process, which included a review of literature and discussion with experts (PAP) and expert by experience (PPI panel), we have not followed a framework such as a Delphi approach. Where there was a disagreement, this was resolved through discussion and consensus; however, a lack of a robust approach like Delphi is a noted limitation of the study. Using routinely collected data sources which capture records using Read codes does present with limitations. There are challenges relating to data quality, degree of data capture, variability in clinician and practice usage, and degree of coverage.^43 44^

In the cohort, a larger proportion of patients live in the most deprived areas of Wales (22.30%), with fewer individuals in less deprived categories. Similar findings have been derived from primary care databases in England.^45^ The cohort has a lack of representation from individuals from non-White ethnic groups. When compared with census data across 2001, 2011 and 2021, percentages of individuals from minority ethnic backgrounds were even lower in the cohort data.^46 47^ Additionally, there was a high percentage (29.68%) of individuals with an unknown ethnic group recording. The data could be strengthened by linking to further data sources available with SAIL in an effort to reduce the unknown ethnic groups.

Research has revealed that the recording of ethnic groups within health data can be of poor quality, where approximately 1 in 10 electronic healthcare records does not have ethnic group information.^48^ People from ethnic minority groups are more likely to have incomplete codes, other codes, or inconsistent and thus unreliable codes. This can lead to the underrepresentation of minority groups, and overrepresentation of ‘other’ ethnic groups codes, masking potential disparities related to ethnic groups.^49^ The UK Health Data Research Alliance Ethnicity Coding Standards Special Interest Group have recently drafted recommendations as a call to action to standardise and optimise ethnic group data recording across health data.^50^ Further work is required to observe a population that adequately represents individuals from a minority ethnic background, and with caution that coding bias is considered when interpreting the relevant data.

The study provides a springboard for interested parties to determine where existing health policies may not suit the needs of individuals with intellectual disability, in an effort to reduce existing health inequalities. Further research should centre around the translation of the findings of this study into actionable insights. Long-term condition clusters, trajectories, outcomes and risk factors should be explored in order to optimise the understanding and longitudinal care of individuals with intellectual disabilities and long-term conditions.

## Data Availability

Data may be obtained from a third party and are not publicly available.
