# Supplementary material for: Nature and prevalence of long-term conditions in people with intellectual disability: retrospective longitudinal population-based study
Source: BMJ Open. 2025 Jan 22;15(1):e090857. doi: 10.1136/bmjopen-2024-090857 (PMC11784237; doi:10.1136/bmjopen-2024-090857)
Supplement: Supplementary file 1 [file bmjopen-2024-090857.reviewer_comments.pdf]

PEER REVIEW HISTORY

BMJ Open publishes all reviews undertaken for accepted manuscripts. Reviewers are asked to complete a checklist review form and are provided with free text boxes to elaborate on their assessment. These free text comments are reproduced below.

ARTICLE DETAILS

Title (Provisional)

Nature and Prevalence of Long-Term Conditions in People with Intellectual Disability: Retrospective Longitudinal Population-Based Study

Authors

Lewin, Gemma; Kousovista, Rania; Abakasanga, Emeka; Shivamurthy, Rishika; Cosma, Georgina; Jun, Gyuchan; Kaur, Navjot; Akbari, Ashley; Gangadharan, Satheesh

---

VERSION 1 - REVIEW

Reviewer 1

Name Roy, Ashok

Affiliation Coventry and Warwickshire Partnership NHS Trust, Psychiatry of Intellectual Disability

Date 06-Oct-2024

COI None

This is an important study looking a the prevalence of long term conditions in the population with intellectual disability in Wales. They authors have studies a large sample producing generalisable findings. However I have a couple issues i need clarification on

1. There is not mention of ethical approval.

2. On line 51 on page 11 the authors lump a number of conditions such as GORD and dyspepsia under gastrointestinal disorders and yet on page 16 line 26 they present finding for reflux disorders.

Reviewer 2

Name Lunsky, Yona

Affiliation Centre for Addiction and Mental Health, Underserved Populations Program

Date 04-Nov-2024

COI **None**

The abstract is clear

Introduction is concise and thorough.

1. There is a statement about health problems related to smoking, alcohol consumption and illicit drug use being uncommon. The citation is from 2006. I think it is an older reference and I think the research is uncommon. But there are a few population based studies. Work from Canada suggests similar or higher rates of addictions (Lin et al https://pubmed.ncbi.nlm.nih.gov/27591020/.) and a recent systematic review supports this as well. https://pubmed.ncbi.nlm.nih.gov/31330514/).

Methods

2. It would be helpful in study design to indicate where information came from on presence of intellectual disability. Which codes were included and did they all come GP records or could they also come from hospitalizations or specialist records? could they come from social services records? Is there a mandatory field on the presence of ID? Have other studies looked at the ID cohort using SAIL databank?

3. I believe that Wales has community based learning disability teams. This is not the case in other countries. Do we know whether the people identified would be tied to LD teams? Perhaps a sentence at the start of methods explaining how people with ID would be served from this study would be helpful (specialized and general health services).

Results

4. It is important to highlight that the Wales population is primarily white, as the authors have done. It is also interesting that the people in the most deprived category is not much higher than 20%. In Ontario, the difference is more evident.

5. The proportion of people with at least one long term condition is almost the entire population.

6. In selecting the final list of MLTC, it would be relevant to note how many conditions were added to the existing list cited by Ho et al. Were any conditions removed? Perhaps this could be indicated in the table of conditions by italicizing the ones that were added.

7. The results and discussion were written a bit differently than I would typically see. The authors do some interpreting and contextualizing of results in the results section which I would typically see in the discussion. I am also used to seeing a summary paragraph at the start of the discussion which highlights what was found and what it means. In this way the discussion starts with summarizing what is most important. In the current discussion, the first thing discussed focuses on how the team selected long term conditions to be studied. I don't think I would start the discussion this way. The second paragraph reads more like what

I might see in the first paragraph. The remainder of the discussion is mostly about strengths and limitations.

I might move some of the contextualizing from the results into the discussion, and I would condense the strengths and limitations - they are still valid but do not need as much text.

VERSION 1 - AUTHOR RESPONSE

Reviewer: 1
1. There is not mention of ethical approval.

Answered as above.

2. *On line 51 on page 11 the authors lump a number of conditions such as GORD and dyspepsia under gastrointestinal disorders and yet on page 16 line 26 they present finding for reflux disorders.*

Thank you for your identification of this matter. This group has been amended to upper gastrointestinal disorders. The findings for reflux disorders had included all codes placed in the gastrointestinal disorders group, however had taken on the name of reflux disorders during data interpretation. All data is now consistent and named upper gastrointestinal disorders for clarity.

Reviewer: 2

1. There is a statement about health problems related to smoking, alcohol consumption and illicit drug use being uncommon. The citation is from 2006. I think it is an older reference and I think the research is uncommon. But there are a few population based studies. Work from Canada suggests similar or higher rates of addictions (Lin et al https://pubmed.ncbi.nlm.nih.gov/27591020/.) and a recent systematic review supports this as well. https://pubmed.ncbi.nlm.nih.gov/31330514/).

Thank you for pointing this out.
This statement has been omitted from the introduction. This was considered following review of the supporting literature. The above systematic review was limited to individuals with mild and borderline intellectual disability which may not reflect the population, and as this is outside of the direct scope of the aims of the study it has been removed to avoid confusion or disinformation.

2. It would be helpful in study design to indicate where information came from on presence of intellectual disability. Which codes were included and did they all come GP records or could they also come from hospitalizations or specialist records? could they come from social services records? Is there a mandatory field on the presence of ID? Have other studies looked at the ID cohort using SAIL databank?

Thank you for your suggestion. There is no mandatory field to record intellectual disability in GP records. To identify the presence of intellectual disability, Read codes from primary care records were used. These codes included codes referring to the presence of intellectual disability or genetic syndromes suggesting the presence of intellectual disability (e.g. Lesch Nyhan Syndrome). A list of Read codes was prepared using previously used repositories as a springboard (https://www.ncbi.nlm.nih.gov/books/NBK453413/) which was then finalised by a Professional Advisory Panel (PAP) which included two Consultant Psychiatrists specialised in Intellectual Disability and two GPs.

Previous research has used Read codes to identify people with intellectual disability from SAIL (Kennedy N, Kennedy J, Kerr M, et al Health checks for adults with intellectual disability and association with survival rates: a linked electronic records matched cohort study in Wales, UKBMJ Open 2022;12:e049441. doi: 10.1136/bmjopen-2021-049441).

3. I believe that Wales has community based learning disability teams. This is not the case in other countries. Do we know whether the people identified would be tied to LD teams? Perhaps a sentence at the start of methods explaining how people with ID would be served from this study would be helpful (specialized and general health services).

Thank you for this comment.
As this is a population that has been extracted from GP Read codes data, they would not necessarily have any Community Learning Disability team input and so information relating to Community teams may not be relevant to them.

4. It is important to highlight that the Wales population is primarily white, as the authors have done. It is also interesting that the people in the most deprived category is not much higher than 20%. In Ontario, the difference is more evident.

We acknowledge the comment
No specific amends.

5. The proportion of people with at least one long term condition is almost the entire population.

We acknowledge the comment.
No specific amends.

6. In selecting the final list of MLTC, it would be relevant to note how many conditions were added to the existing list cited by Ho et al. Were any conditions removed? Perhaps this could be indicated in the table of conditions by italicizing the ones that were added.

Thank you for this suggestion.

This has now been indicated in the table of conditions with italicization for clarity. There were changes such as conditions removed where a wider group of conditions would be grouped together, which would be extensive to explain in-text and result from multiple PAP and PPI meetings. The added conditions amount to 12 and result from either a new grouping of multiple conditions, or conditions which are of importance to individuals with intellectual disability.

7. The results and discussion were written a bit differently than I would typically see. The authors do some interpreting and contextualizing of results in the results section which I would typically see in the discussion. I am also used to seeing a summary paragraph at the start of the discussion which highlights what was found and what it means. In this way the discussion starts with summarizing what is most important. In the current discussion, the first thing discussed focuses on how the team selected long term conditions to be studied. I don't think I would start the discussion this way. The second paragraph reads more like what I might see in the first paragraph. The remainder of the discussion is mostly about strengths and limitations.

I might move some of the contextualizing from the results into the discussion, and I would condense the strengths and limitations - they are still valid but do not need as much text.

Thank you for your suggestion. We have amended the results section so that it is more descriptive and have moved contextualisation into the discussion section. Hopefully this better demonstrates the findings and utility of the study and improves reading experience.

VERSION 2 - REVIEW

**Reviewer**	**2**
**Name**	**Lunsky, Yona**
**Affiliation**	**Centre for Addiction and Mental Health, Underserved Populations Program**
**Date**	**17-Dec-2024**
**COI**	

I think this is an important contribution to the literature and I appreciate the restructuring of the results/discussion. I also like the highlights of unique long term conditions for this group.
